# Common garden experiments reveal uncommon responses across temperatures, locations, and species of ants

**DOI:** 10.1002/ece3.407

**Published:** 2012-11-02

**Authors:** Shannon L Pelini, Sarah E Diamond, Heidi MacLean, Aaron M Ellison, Nicholas J Gotelli, Nathan J Sanders, Robert R Dunn

**Affiliations:** 1 Harvard Forest, Harvard UniversityPetersham, Massachusetts, 01366; 2 Department of Biological Sciences, Bowling Green State UniversityBowling Green, Ohio, 43403; 3 Department of Biology, North Carolina State UniversityRaleigh, North Carolina, 27695; 4 Department of Biology, University of North CarolinaChapel Hill, North Carolina, 27599; 5 Department of Biology, University of VermontBurlington, Vermont, 05405; 6 Department of Ecology and Evolutionary Biology, University of TennesseeKnoxville, Tennessee, 37996

**Keywords:** Climate change, common garden, Formicidae, interspecies variation, intraspecies variation, warming experiment

## Abstract

Population changes and shifts in geographic range boundaries induced by climate change have been documented for many insect species. On the basis of such studies, ecological forecasting models predict that, in the absence of dispersal and resource barriers, many species will exhibit large shifts in abundance and geographic range in response to warming. However, species are composed of individual populations, which may be subject to different selection pressures and therefore may be differentially responsive to environmental change. Asystematic responses across populations and species to warming will alter ecological communities differently across space. Common garden experiments can provide a more mechanistic understanding of the causes of compositional and spatial variation in responses to warming. Such experiments are useful for determining if geographically separated populations and co-occurring species respond differently to warming, and they provide the opportunity to compare effects of warming on fitness (survivorship and reproduction). We exposed colonies of two common ant species in the eastern United States, *Aphaenogaster rudis* and *Temnothorax curvispinosus*, collected along a latitudinal gradient from Massachusetts to North Carolina, to growth chamber treatments that simulated current and projected temperatures in central Massachusetts and central North Carolina within the next century. Regardless of source location, colonies of *A. rudis*, a keystone seed disperser, experienced high mortality and low brood production in the warmest temperature treatment. Colonies of *T. curvispinosus* from cooler locations experienced increased mortality in the warmest rearing temperatures, but colonies from the warmest locales did not. Our results suggest that populations of some common species may exhibit uniform declines in response to warming across their geographic ranges, whereas other species will respond differently to warming in different parts of their geographic ranges. Our results suggest that differential responses of populations and species must be incorporated into projections of range shifts in a changing climate.

## Introduction

The majority of forecasts of the responses of species to climatic warming assume that populations within a species are homogeneous and thus model shifts in the geographic distributions of entire species (but see Buckley 2008; Richardson et al. 2011 for exceptions). Likewise, most empirical studies of recent responses to warming focus on individual species and/or locations (but see Pelini et al. 2011) as invariant units of analysis. However, the rate, magnitude, and direction of the responses to warming or other climatic changes by different individuals in different populations of any given species may differ for at least two reasons. First, populations may be locally adapted to current or historical environmental conditions (Gilman et al. 2006; Pelini et al. 2009; Angert et al. 2011). Second, individuals from different populations may differ in their ability to cope with local environmental changes (Magnani 2009). For these reasons, models based on the assumption of uniform responses among populations within a species may be misleading.

The methods necessary to assess if populations are locally adapted to climate or can cope with, or even benefit from climatic change are well established (reviewed in Kawecki and Ebert 2004). The first step is to determine experimentally whether and how individuals from distinct populations vary in their ability to respond to common conditions (Grosholz 2001; Castañeda et al. 2005; Pelini et al. 2009; Tack and Roslin 2010; Craig et al. 2011). The second step is to conduct common garden experiments with treatments that represent different climatic regimes. Three broad outcomes are possible. First, all populations might exhibit increased survivorship or reproduction in response to warming. Second, populations might exhibit local adaptation to historical conditions, or have narrow physiological tolerances, thereby leading to population declines or extinctions under warming. Third, local populations may respond idiosyncratically to warming, with some populations exhibiting local adaptation/narrow physiological tolerances and declining in response to temperature increases, whereas other populations cope with and/or increase in response to temperature increases. All these outcomes are possible because individuals are behaviorally or phenotypically plastic and populations of individuals possess genetic variation in traits that maximize fitness for different individuals in different conditions.

In this study, we used ants to examine variation among populations and co-occurring species under expected temperature change in the eastern United States (also see Fitzpatrick et al. 2011; Jenkins et al. 2011). Ants are an ideal taxon to use for multiple common-garden experiments because they are responsive to temperature (Dunn et al. 2009) and relatively easy to maintain in controlled environments. Temperature is correlated with patterns of ant diversity and abundance (Sanders et al. 2007), seasonal patterns of activity (Dunn et al. 2007), overwintering mortality (Sorvari et al. 2011), foraging behavior (Ruano et al. 2000), and the outcomes of interactions between species (Cerda et al. 1997; Holway et al. 2002). Ant foraging activities modulate many ecosystem processes, including decomposition, nutrient cycling, and primary production (Hölldobler and Wilson 1990; Folgarait 1998; Del Toro et al. 2012). Consequently, the extent to which ants respond to climatic change, especially to local and regional changes in temperature, may have cascading consequences for other taxa and for ecosystem dynamics (Lensing and Wise 2006; Moya-Larano and Wise 2007). Another study has demonstrated that ant community responses to warming differ across latitude (Pelini et al. 2011), making ants an ideal taxon for examining the underlying causes of geographic variation in the ecological responses to climate change.

Using a common garden experiment, we tested the hypothesis that the relationship between temperature and fitness will vary for ant populations sampled across a species' range. In order to understand if patterns in intraspecies variation in temperature impacts on fitness are generalizable, we tested the hypothesis that co-occurring, closely related species with similar geographic distributions would display similar patterns in intraspecies variation in their response to varying temperature; this is one of the first studies to experimentally test this hypothesis. Growth chamber studies are particularly useful for examining insect responses to warming because they circumvent heat-island effects associated with warming treatments applied in the field (Moise and Henry 2010). We placed ant colonies in growth chambers set to summer temperatures in the regions from which ants were collected as well as to mimic summer temperatures in the future (Solomon 2007). To determine whether ants from different climates differed in their ability to cope with shifts in temperature, and more generally to determine whether warming could have a net negative or positive effect on populations across the geographic ranges of species, we examined associations between source-location mean summer temperature and experimental rearing temperature on two measures of fitness: survival and brood production. Fitness differences attributed to source-location temperatures would suggest that individuals from different locations differed in their ability to cope with temperature change. Increases in fitness with increases in rearing temperature would suggest that populations throughout species' ranges will have increased fitness under warming, whereas decreases would suggest that warming will have negative fitness effects across species' ranges. Fitness differences attributed to interactions between source-location temperatures and rearing temperature would suggest that populations from different locales are affected differently by temperature shifts.

## Methods

### Common garden

The focal taxa for these experiments were populations of *Aphaenogaster rudis* Mayr and *Temnothorax curvispinosus* Mayr from Massachusetts to North Carolina (33.6–42.5°; Table 1). These two ant species co-occur across forests in the eastern United States (Pelini et al. 2011). While *T. curvispinosus* is recognized as a species (Mackay 2000), *A. rudis* is a species complex (Umphrey 1996) currently undergoing taxonomic revision (Bernice DeMarco, unpubl. data). In order to determine whether patterns observed in our focal species were similar to those for other species, we also included a subset of colonies of other species that co-occur with the focal species: *A. fulva* Roger, *Camponotus chromaiodes* Bolton, *Crematogaster lineolata* Say, *Tapinoma sessile* Say, and *Temnothorax longispinosus* Roger (Table 1). We placed single queen colonies in artificial nest boxes and allowed them to acclimate to laboratory conditions for 2 weeks before placing them into growth chambers at North Carolina State University laboratory facilities. Artificial nests were plastic containers (390 cm^3^) with sand, water tubes plugged with cotton (to maintain humidity), and a food source (Bhatkar and Whitcomb 1970). When brood or males were collected with the colonies, we removed them so as to assess more accurately survival and reproductive output of the colony throughout the duration of the experiment.

**Table 1:** Source locations (decimal degrees), mean summer temperatures (WorldClim, Hijmans et al. 2005) at source locations, and number of colonies placed into three growth chamber temperature treatments from each species

			Rearing temperature (# colonies)		
			
Species	Source locations (decimal degrees)	Mean summer temperature (°C)	21°C	26°C	31°C
*Aphaenogaster rudis*	33.63°, −91.79°	26.1	1	2	2
	35.78°, −78.80°	24.8	0	1	0
	36.04°, −79.07°	24.1	5	4	5
	39.89°, −74.58°	22.3	0	1	0
	40.02°, −83.01°	22.1	0	0	1
	42.53°, −72.19°	18.5	4	4	4
*Temnothorax curvispinosus*	35.76°, −78.68°	24.8	12	11	12
	38.57°, −77.37°	23.7	0	1	1
	39.64°, −74.66°	22.6	0	0	1
	40.44°, −74.27°	22.4	1	1	1
	41.84°, −70.67°	20.4	2	2	3
	42.35°, −72.19°	18.5	2	2	2
*Aphaenogaster carolinenesis*	35.78°, −78.68°	24.7	0	0	1
	38.51°, −90.83°	23.8	0	0	1
*Aphaenogaster fulva*	38.51°, −90.83°	23.8	1	1	1
*Camponotus chromaiodes*	38.51°, −90.83°	23.8	0	1	1
*Crematogaster lineolata*	36.04°, −79.07°	24.1	0	1	1
	40.58°, −76.75°	21.2	0	1	1
	42.53°, −72.19°	18.5	0	1	0
*Tapinoma sessile*	38.51°, −90.83°	23.8	0	0	1
	40.02°, −83.01°	22.1	1	1	2
*Temnothorax longispinosus*	42.53°, −72.19°	18.5	2	1	2

For species × sampling locations with fewer than three colonies, priority was given to the intermediate (26°C) and warmest (31°C) temperature treatments. Black text indicates focal species; nonfocal species are gray.

We placed colonies in their artificial nest boxes into one of three growth chamber temperature treatments, with temperatures determined from long-term temperature records from Harvard Forest, Massachusetts (21°C summer mean); Duke Forest, North Carolina (26°C summer mean) and Miami, Florida (31°C summer mean); the 26°C treatment represents projected warming for Massachusetts before 2100, and the warmest treatment, 31°C, represents the forecast temperature for Massachusetts beyond 2100 and for North Carolina before 2100 (Solomon 2007). Chamber temperatures fluctuated diurnally, that is, temperatures were ramped up/down by 1.2°C per hour between the average minimum (at 3 am) and maximum (at 3 pm) temperatures for each location (Massachusetts: 16–26°C; North Carolina: 21–31°C; Florida: 26–36°C), and day-length was 14 h long in all chambers.

We checked nests daily to ensure constant water and food supply. We censused ant colonies in July before transferring them to the growth chambers, again 10 days after the start of the experiment, and finally at the end of the experiment in September (59 days total). At each census, we recorded the presence or absence of brood and the number of workers in each colony.

### Data analysis

First, we used generalized linear mixed models (R version 2.9.0; R Development Core Team 2011) to test whether survival and brood production (binomial response variables) were significantly affected by source-location temperature (fixed effect) and/or rearing temperature (fixed effect), across all species (random effect), and both census periods (random effect). To determine whether patterns found across our entire species pool were consistent with those for the focal species, *A. rudis* and *T. curvispinosus*, for which we had broader geographic coverage, we ran similar models examining the effects of source-location temperature, rearing temperature, and species as fixed effects, and census period as a random effect. We also included a species × source-location temperature term to determine whether the ability of colonies from different source locations to cope with temperature change was similar in both focal species. In addition, we also included a species × rearing temperature term in this model to determine whether the two focal species differed in their responses to rearing temperature, regardless of source location. Finally, because both species × source-location temperature and species × rearing temperature had significant effects on survival of the two focal species, we examined in more detail the separate responses of *A. rudis* and *T. curvispinosus*. For each of these two species, we modeled survival as a function of source-location temperature, rearing temperature and their interaction. Significant interactions between source-location temperature and rearing temperature revealed if colonies from different locales were affected differently by similar temperatures, which may be due to adaptive differences, such as local adaptation. We extracted the mean summer (warmest quarter) temperatures at the source locations from WorldClim (Hijmans et al. 2005).

## Results

### Interspecies models

**Figure 1:**
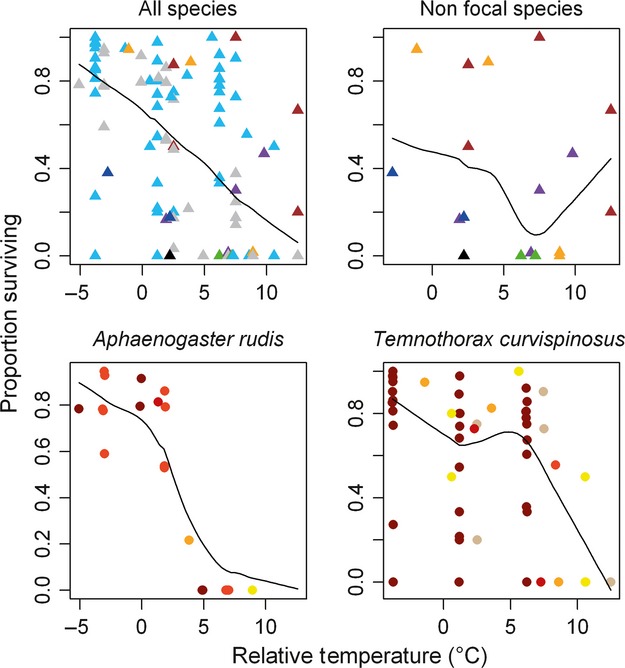
Scatterplots of survival (at final census) as a function of mean rearing temperature – source-location summer temperatures. Upper left panel shows data for all species considered in the experiment; upper right shows all species except for *Aphaenogaster rudis* and *Temnothorax curvispinosus*; lower left shows *A. rudis*; and lower right shows *T. curvispinosus* survival. Positive *x*-axis values indicate cases when experimental temperatures were higher than those at colony source locations, and negative values indicate cases when rearing temperatures were lower. Lines represent locally weighted scatterplot smoothing (function loess in R). In plots with multiple species (upper panels), species are shown in different colors: green, *Aphaenogaster carolinenesis*; blue *A. fulva*; gray, *A. rudis*; black, *Camponotus chromaiodes*; purple, *Crematogaster lineolata*; orange, *Tapinoma sessile*; light blue, *Temnothorax curvispinosus*; brown, *Temnothorax longispinosus*. For *A. rudis* and *T. curvispinosus* (bottom panels), colors represent mean summer temperatures at source locations: *A. rudis* – dark red, 26.1°C; red, 24.8°C; orange-red, 24.1°C; orange, 22.3°C; yellow, 22.1°C; tan, 18.5°C; *T. curvispinosus* – dark red, 24.8°C; red, 23.7°C; orange-red, 22.6°C; orange, 22.4°C; yellow, 20.4°C; tan, 18.5°C.

Survival decreased with increasing rearing temperature (all species: χ^2^ = 5800; *P* < 0.001; focal species: χ^2^ = 3800; *P* < 0.001), but increased with source-location temperature (all species: χ^2^ = 4.3; *P* = 0.037; focal species: χ^2^ = 500; *P* < 0.001) (Fig. 1). Brood production also decreased with increasing rearing temperature (all species: χ^2^ = 19; *P* < 0.001; focal species: χ^2^ = 18; *P* < 0.001). In our focal species model of survival, the species × source-location temperature and species × rearing temperature terms also were significant (χ^2^ = 1900; *P* < 0.001; χ^2^ = 6000; *P* < 0.001, respectively).

### Intraspecies models

**Figure 2:**
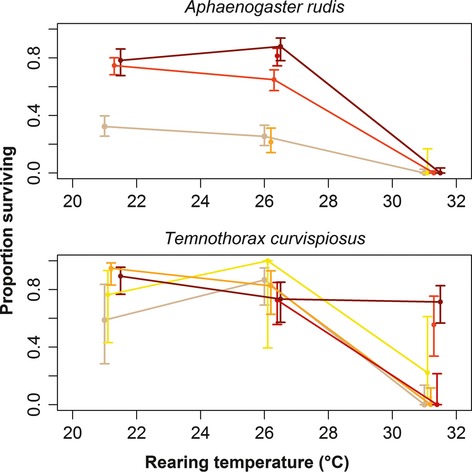
Mean survival (at final census) of *Aphaenogaster rudis* (upper panel) and *Temnothorax curvispinosus* (lower panel) as a function of rearing temperature. Error bars represent 95% binomial proportion confidence intervals. Lines are colored by source locations such that the coolest location is tan and the warmest is dark red. Colors represent mean summer temperatures at source locations: *A. rudis* – dark red, 26.1°C; orange-red, 24.1°C; tan, 18.5°C; *T. curvispinosus* – dark red, 24.8°C; red, 23.7°C; orange, 22.4°C; yellow, 20.4°C; tan, 18.5°C. Points are jittered along the *x*-axis so that points of overlap between different source locations can be visible. Rearing temperatures were 21°C, 26°C, and 31°C.

Survival of both *A. rudis* and *T. curvispinosus* decreased with increasing rearing temperature (χ^2^ = 120; *P* < 0.001 and χ^2^ = 36; *P* < 0.001, respectively), but increased with source-location temperature (χ^2^ = 200; *P* < 0.001 and χ^2^ = 17; *P* < 0.001, respectively) (Fig. 1, lower panels). Interactions between source-location and rearing temperature also were significant, but different, for both species (*A. rudis*: χ^2^ = 270; *P* < 0.001 and *T. curvispinosus*: χ^2^ = 30; *P* < 0.001). More specifically, *A. rudis* colonies from warmer locations had higher survival than those from cooler locations in the low and intermediate rearing temperatures, but all colonies had high mortality in the warmest rearing temperature (Fig. 2, upper panel). In contrast, *T. curvispinosus* colonies from different source locations did not differ significantly in survival except in the warmest rearing temperature, where colonies from two of the three warmest source locations had relatively high survival compared with their cooler source-location counterparts (Fig. 2, lower panel).

## Discussion

Species are composed of individual populations, which may be subject to different selection pressures. Some will go extinct locally or globally, some will migrate, and some will increase in size (Pelini et al. 2009). Increasing temperatures may have negative fitness effects for populations that are locally adapted to and/or have narrow physiological tolerances of temperature and positive fitness effects for other populations with broader physiological tolerances of temperature. If different populations respond differently to climatic warming, then extrapolating to a single, overall response of the given species may be unwise or unwarranted. Furthermore, a species' potential to adapt to future climatic change may be reduced if some populations perform well, whereas others decline under warming and causes a reduction in genetic diversity (Collevatti 2011). In aggregate (at the species level), all eight ant species that we studied in this common garden experiment exhibited decreased survival and brood production with increased warming. However, we observed strong differences among species and populations within particular species. Colonies of both focal species, *T. curvispinosus* and *A. rudis*, from warmer locales had higher survival and brood production under warmer temperatures than those from cooler sites. Survival decreased with increasing temperatures for *A. rudis* from all locales. The results for *T. curvispinosus* were very similar, with one exception: colonies of *T. curvispinosus* from the warmest locale experienced increased fitness in the warmer temperatures. Together, these findings suggest that for many of the species in our study system, warming may be detrimental. However, where responses differ among populations within species, warming affects southern populations (from warmer climates) less than it does northern populations (from cooler climates). This latter result suggests that forecasted distributions of ant species in a warmed world, whether based on physiology or distributional data, do not account for intraspecific variability and may be inaccurate.

Because *A. rudis* populations responded negatively to temperature increases regardless of their location of origin, we forecast that severe warming will negatively affect populations of this species across its entire range. As the primary disperser of many forest understory herbs (Ness et al. 2009), reductions in *Aphaenogaster* populations are likely to have ramifying consequences in many forests (e.g., Rodriguez-Cabal et al. 2012). Unlike *A. rudis*, *T. curvispinosus* colonies from warmer, southern locales performed well under warming, whereas their counterparts from cooler, northern locales did not. Southern *T. curvispinosus* populations may have more genetic diversity in traits related to physiological tolerances than northern colonies. We also observed noticeably increased foraging activity in *A. rudis* (S. Diamond, pers. obs.) and running speeds in *T. curvispinosus* (H. MacLean, unpubl. data) in the warmer temperature treatments, suggesting that some of the mortality associated with warming may be due to changes in behavioral and physiological traits (e.g., Dillon et al. 2010).

The findings from this laboratory common garden experiment complement those from recent field warming manipulations in the same system with many of the same ant species. In field warming experiments, we found increases in abundance under warming up to 5°C for low latitude (North Carolina) populations of species with higher thermal tolerances, including *C. lineolata* (and see Pelini et al. 2011) and *T. curvispinosus*, but not for *A. rudis* and other species with lower thermal limits. Abundances of species at a higher latitude site (Massachusetts) increased under warming regardless of their thermal tolerances (Diamond et al. In press). The data from the laboratory common garden experiment reported here, together with data from our previous field experiments, suggest that the responses of ants to warming will vary across populations within and across species (also see Fitzpatrick et al. 2011; Jenkins et al. 2011 for modeling of ant communities under climate change).

Future studies should address how such changes could have cascading consequences for species interactions and ecosystem processes (Traill et al. 2010) that are localized and are not well projected by current models that assume uniform responses of species across their entire range. Multiple observational and experimental approaches should be integrated because complex abiotic (e.g., humidity, rainfall) and biotic (e.g., interactions with predators/prey or plants) changes associated with climatic change can be captured by field manipulations, but separated by common garden laboratory experiments.
